# PyLandStats: An open-source Pythonic library to compute landscape metrics

**DOI:** 10.1371/journal.pone.0225734

**Published:** 2019-12-05

**Authors:** Martí Bosch

**Affiliations:** Urban and Regional Planning Community (CEAT), École Polytechnique Fédérale de Lausanne (EPFL), Lausanne, Switzerland; Universita degli Studi di Trento, ITALY

## Abstract

Quantifying the spatial pattern of landscapes has become a common task of many studies in landscape ecology. Most of the existing software to compute landscape metrics is not well suited to be used in interactive environments such as Jupyter notebooks nor to be included as part of automated computational workflows. This article presents PyLandStats, an open-source Pythonic library to compute landscape metrics within the scientific Python stack. The PyLandStats package provides a set of methods to quantify landscape patterns, such as the analysis of the spatiotemporal patterns of land use/land cover change or zonal analysis. The implementation is based on the prevailing Python libraries for geospatial data analysis in a way that they can be forthwith integrated into complex computational workflows. Notably, the provided methods offer a large variety of options so that users can employ PyLandStats in the way that best supports their needs. The source code is publicly available, and is organized in a modular object-oriented structure that enhances its maintainability and extensibility.

## Introduction

Landscape ecology is based on the notion that the spatial pattern of landscapes strongly influences the ecological processes that occur upon them [1]. From this perspective, quantifying the spatial patterns of landscapes becomes a central prerequisite to the study of the pattern-process relationships. Landscape ecologists often view landscapes as an heterogeneous spatial mosaic of discrete patches, each representing a zone of relatively homogeneous conditions, where the size, shape and configuration of patches significantly affects key ecosystem functions such as biodiversity and fluxes of organisms and materials [2].

Recent decades have seen the development of a series of landscape metrics that quantify several aspects of the spatial pattern of landscapes [3–5]. In a context of significant advances in geographical information systems (GIS) and increasing availability of land use/land cover (LULC) datasets, landscape metrics have been implemented within a variety of software packages [6]. The present article introduces PyLandStats, an open-source library to compute landscape metrics, which represents an advance over previously available software because of its implementation within the most popular libraries of the scientific and data-centric Python stack. Additionally, its modular and object-oriented design allows it to be efficiently used in interactive environments such as Jupyter notebooks as well as in automated computational workflows, and eases the maintainability and extensibility of the code.

The remainder of the article describes the structure and use of PyLandStats by presenting a thorough example analysis case for a sequence of three raster landscape snapshots of the Canton of Vaud (Switzerland) for the years 2000, 2006 and 2012, which have been extracted from the Corine Land Cover [7] inventory. The code snippets and materials to reproduce the figures of the following four sections can be found in S1, S2, S3 and S4 Codes respectively.

## Analysis of a single landscape

The basic unit of the PyLandStats library is the Landscape class, which represents the LULC mosaic of a particular region *at a given point in time*. A Landscape instance mainly consists of an array where each position represents the LULC class at the corresponding pixel of the lanscape.

Since LULC data is most often stored in raster files (e.g., GeoTiff), the easiest way to instantiate a Landscape object is by passing a path to a raster file as first argument, as in:

> ls = Landscape(‘*path/to/raster.tif*’)

The above call will use the rasterio Python library in order to read the raster files, and will extract the pixel resolution and no-data value from the file metadata. Alternatively, Landscape instances might also be initialized by passing a NumPy array [8] as first argument, which also requires specifying the x and y coordinates of pixel resolution as a tuple in the res keyword argument. By default, PyLandStats assumes that zero values in the array represent pixels with no data. Otherwise, the no-data value can be specified by means of the nodata keyword argument. A Landscape instance can be plotted at any moment by using its plot_landscape method. Note that all the plotting methods of PyLandStats make use of the matplotlib library [9].

### Computing data frames of landscape metrics

Landscape metrics might be classified into two main groups (see S1 Table for the list of metrics implemented in PyLandStats, their classification and their description). The first concerns metrics that provide a scalar value for each patch of the landscape, which are often referred to as patch-level metrics. The second consists of metrics that provide a scalar value that aggregates a characteristic of interest over a set of the patches. This second group allows for an additional distinction between class-level metrics, which are computed over all patches of a given LULC class, and landscape-level metrics, which are those computed over all the patches of a landscape.

For a given Landscape instance, the patch-level metrics can be computed by means of the compute_patch_metrics_df method as in:

*#* ‘*ls*’ *is a given ‘Landscape’ instance*

> ls.compute_patch_metrics_df()

which will return a pandas data frame [10] as depicted in Table 1, where each row corresponds to a patch of the landscape with its associated LULC class value and the computed metrics.

**Table 1:** Example data frame of patch-level metrics.

patch_id	class_val	area	perimeter	perimeter_area_ratio	shape_index	fractal_dimension	euclidean_nearest_neighbor
0	1	115.0	10600.0	92.173913	2.409091	1.129654	1431.782106
1	1	13.0	2600.0	200.000000	1.625000	1.100096	223.606798
2	1	2.0	600.0	300.000000	1.000000	1.011893	223.606798
⋮	⋮	⋮	⋮	⋮	⋮	⋮	⋮
203	2	11.0	1800.0	163.636364	1.285714	1.052571	223.606798
204	2	2.0	800.0	400.000000	1.333333	1.069990	223.606798
205	2	14.0	2400.0	171.428571	1.500000	1.079705	282.842712

Similarly, metrics can be computed at the class level by using the compute_class_metrics_df method as in:

> ls.compute_class_metrics_df()

which will return a pandas data frame as depicted in Table 2, where each row corresponds to a LULC class and each column represents a metric computed at the row’s class level.

**Table 2:** Example data frame of class-level metrics.

class_val	total_area	proportion_of_landscape	number_of_patches	patch_density	largest_patch_index	total_edge	…
1	24729	7.701939	193	0.060111	2.069921	1431600	…
2	296346	92.298061	13	0.004049	89.451374	1431600	…

Lastly, the landscape-level metrics can be computed by using the compute_landscape_metrics_df method as in:

> ls.compute_landscape_metrics_df()

which will return a pandas data frame as depicted in Table 3, where the only row features the values of the metrics computed at the landscape level.

**Table 3:** Example data frame of landscape-level metrics.

	total_area	number_of_patches	patch_density	largest_patch_index	total_edge	edge_density	landscape_shape_index	…
0	321075	206	0.064159	89.451374	1431600	4.458771	9.716931	…

### Customizing the landscape analysis

While a vast collection of metrics have been proposed over the literature of the last decades, many of them are highly correlated with one another. As a matter of fact, Riitters et al. [11] found that the characteristics represented by 55 prevalent landscape metrics could be reduced to only 6 independent factors. Therefore, analysis cases tend to consider a limited subset of metrics. To that end, the three methods that compute data frames of metrics showcased above can be customized by means of the metrics keyword argument as in:

> ls.compute_class_metrics_df(

 metrics = [‘*proportion_of_landscape*’, ‘*edge_density*’])

which will return a pandas data frame where only the specified metrics will appear as columns.

On the other hand, certain metrics allow for some customization concerning the way in which they are computed. In PyLandStats, each metric is defined in its dedicated method in the Landscape class, which includes metric-specific keyword arguments that allow controlling how the metric is computed. For instance, when computing the edge density (ED), the user might decide whether edges between LULC pixels and no-data pixels (e.g., landscape boundaries) are considered, or whether the area should be converted to hectares. By default, PyLandStats computes the metrics according to the definitions specified in FRAGSTATS v4 [5] (see also S5 Code), and therefore does not consider edges between LULC pixels and no-data pixels, and converts areas to hectares. Nevertheless, the user might decide to change that by providing the count_boundary and hectares keyword arguments to the edge_density method as in:

> ls.edge_density()

4.4587713151132915

> ls.edge_density(count_boundary = True)

6.863816865218407

> ls.edge_density(count_boundary = True, hectares = False)

0.0006863816865218407

Similarly, the compute_patch_metrics_df, compute_class_metrics_df, and compute_landscape_metrics_df accept the metrics_kws keyword argument in the form of a dictionary, which allows setting the keyword arguments that must be passed to each metrics’ method when computing the data frames. For instance, in order to compute a class-level data frame with the proportion_of_landscape as a fraction instead of a percentage, and include the landscape boundaries in edge_density, the metrics_kws keyword argument must be provided as in:

> ls.compute_class_metrics_df(

 metrics_kws={

  ‘*proportion_of_landscape*’: {‘*percent*’: False},

  ‘*edge_density*’: {‘*count_boundary*’: True}

 })

In the above example, the columns of the returned data frame will feature not only the proportion of landscape and edge density, but all the available metrics instead. In order to compute a reduced set of metrics, some of which with non-default arguments, both metrics and metric_kws keyword arguments must be defined. For instance, in the code snippet below:

> ls.compute_class_metrics_df(

 metrics = [

  ‘*proportion_of_landscape*’, ‘*edge_density*’, ‘*fractal_dimension_am*’

 ],

 metrics_kws={

  ‘*proportion_of_landscape*’: {‘*percent*’: False},

  ‘*edge_density*’: {‘*count_boundary*’: True}

 })

the returned data frame will be of the form depicted in Table 4.

**Table 4:** Example of a data frame of class-level metrics computed with custom metrics and metrics_kws keyword arguments.

class_val	proportion_of_landscape	edge_density	fractal_dimension_am
1	0.077019	4.502998	1.129561
2	0.922981	6.819590	1.204003

Note that the metrics and metric_kws keyword arguments work in the same way for the compute_patch_metrics_df and compute_landscape_metrics_df methods. Additionally, a list of LULC class values might be provided to the classes keyword argument of compute_class_metrics_df in order to compute the metrics for the specified subset of classes only. The three keyword arguments are complimentary and might therefore be used in conjunction. For instance, adding a classes = [1] to the foregoing code snippet would return a data frame of the form depicted in Table 4 but featuring only the first row.

## Spatiotemporal analysis

Landscape metrics are often applied to assess the spatiotemporal patterns of LULC change for a given region by computing landscape metrics over a temporally-ordered sequence of landscape snapshots. To this end, PyLandStats features the SpatioTemporalAnalysis class, which is instantiated with a temporally-ordered sequence of landscape snapshots.

> input_filepaths = [

  ‘*snapshot00.tif*’, ‘*snapshot06.tif*’, ‘*snapshot12.tif*’

 ]

> dates = [2000, 2006, 2012] *# the dates of each snapshot*

> sta = pls.SpatioTemporalAnalysis(input_filepaths, dates = dates)

When initializing a SpatioTemporalAnalysis instance, a Landscape instance will be created for each of the landscape snapshots provided as first argument. The dates argument might also be provided as string or datetime objects (see S2 Code).

### Computing spatiotemporal data frames

Similarly to Landscape instances, the data frames of class and landscape-level metrics of a SpatioTemporalAnalysis instance can be computed by means of the compute_class_metrics_df and compute_landscape_metrics_df methods respectively. For instance, following the snippet above, the data frame of class-level metrics can be obtained as in:

> sta.compute_class_metrics_df()

which will return a data frame indexed by both the class value and date, as depicted in Table 5.

**Table 5:** Example data frame of class-level metrics for a spatiotemporal analysis.

class_val	dates	total_area	proportion_of_landscape	number_of_patches	patch_density	largest_patch_index	total_edge	…
1	2000	24729	7.70194	193	0.0601106	2.06992	1.4316e+06	…
	2006	24599	7.66145	200	0.0622907	2.02227	1.436e+06	…
	2012	24766	7.71346	201	0.0626022	2.02227	1.4459e+06	…
2	2000	296346	92.2981	13	0.0040489	89.4514	1.4316e+06	…
	2006	296476	92.3386	8	0.00249163	89.1318	1.436e+06	…
	2012	296309	92.2865	8	0.00249163	89.0916	1.4459e+06	…

Similarly, the data frame of landscape metrics can be obtained as follows:

> sta.compute_landscape_metrics_df()

where the resulting data frame will be indexed by the dates as depicted in Table 6.

**Table 6:** Example data frame of landscape-level metrics for a spatiotemporal analysis.

dates	total_area	number_of_patches	patch_density	largest_patch_index	total_edge	edge_density	landscape_shape_index	…
2000	321075	206	0.0641595	89.4514	1.4316e+06	4.45877	9.71693	…
2006	321075	208	0.0647824	89.1318	1.436e+06	4.47248	9.73633	…
2012	321075	209	0.0650938	89.0916	1.4459e+06	4.50331	9.77998	…

Note that PyLandStats does not compute data frames for spatiotemporal analyses at the patch level, given that new patches emerge and others disappear over the years and therefore there is no common index upon which the data frames of patch-level metrics for different snapshots could be assembled.

### Customizing the spatiotemporal analysis

As with the Landscape class, the compute_class_metrics_df and compute_landscape_metrics_df methods of the SpatioTemporalAnalysis class also allow customizing how each metric is computed by means of the metrics and metric_kws arguments. Additionally, the classes keyword argument might be provided to compute_class_metrics_df in order to compute the metrics for the specified subset of classes only. For instance, the code snippet below:

> sta.compute_class_metrics_df(

 metrics = [‘*proportion_of_landscape*’, ‘*edge_density*’,

  ‘*fractal_dimension_am*’, ‘*landscape_shape_index*’,

  ‘*shannon_diversity_index*’],

 classes = [1],

 metrics_kws = {

  ‘*proportion_of_landscape*’: {‘*percent*’: False},

  ‘*edge_density*’: {‘*count_boundary*’: True}})

will return a data frame of the form depicted in Table 7.

**Table 7:** Example of a data frame of class-level metrics for a spatiotemporal analysis computed with custom classes, metrics and metrics_kws keyword arguments.

class_val	dates	edge_density	fractal_dimension_am	landscape_shape_index	proportion_of_landscape
1	2000	4.503	1.12956	22.9492	0.0770194
	2006	4.51608	1.12336	23.0892	0.0766145
	2012	4.54847	1.12347	23.181	0.0771346

Note that although provided within the metrics keyword argument, the Shannon’s diversity index does not appear in the data frame of Table 7 since it can only be computed at the landscape level. Analogously, the proportion of landscape would not appear in the data frame of landscape-level metrics.

### Plotting the evolution of metrics

One of the most important features of the SpatioTemporalAnalysis class is plotting the evolution of the metrics. To that end, the class features the plot_metric method, which takes the snake case label of the respective metric name as first argument, e.g., for proportion of landscape, the argument becomes ’proportion_of_landscape’ (see S1 Table for the list of metrics implemented in PyLandStats and their respective snake case labels). In order to plot the evolution of a metric at the class level, the value of the LULC class must be passed to the class_val keyword argument as in:

# a class value of 1 represents “urban” LULC in this example

**Fig 1:**
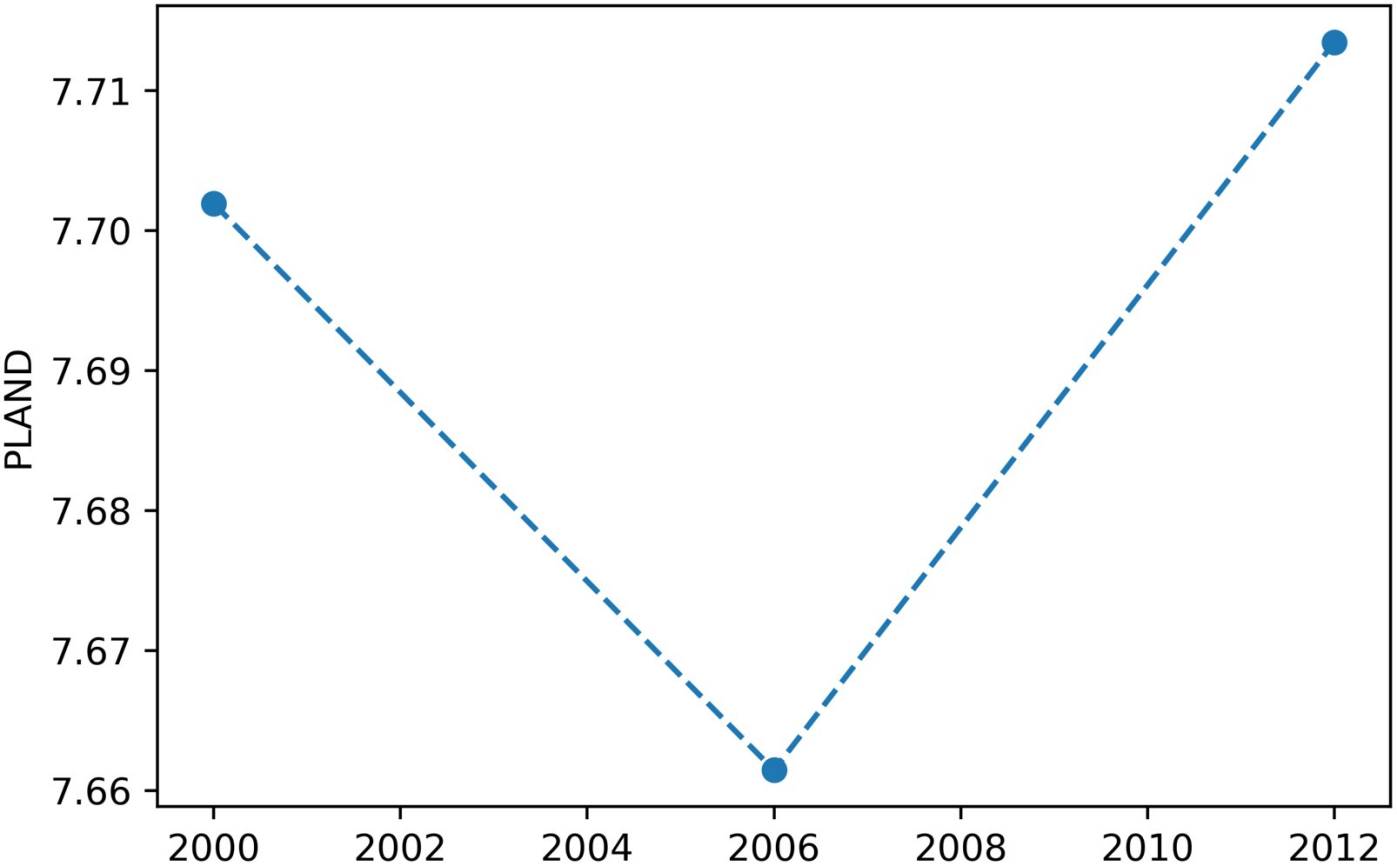
Example of a plot for a class-level metric in a spatiotemporal analysis.

> sta.plot_metric(‘*proportion_of_landscape*’, class_val = 1)

which will produce a plot for the metric at the class level as depicted in Fig 1.

If the class_val keyword argument is ommited, the metric will instead be plotted at the landscape level. For instance, the following snippet will plot both the class and landscape-level area-weighted fractal dimension in the same matplotlib axis:

> ax = sta.plot_metric(‘*fractal_dimension_am*’, class_val = 1,

         plot_kws={‘*label*’: ‘*class level* (*urban*)’})

> sta.plot metric(

  ‘*fractal_dimension_am*’, ax = ax, plot_kws={‘*label*’: ‘*landscape level*’})

**Fig 2:**
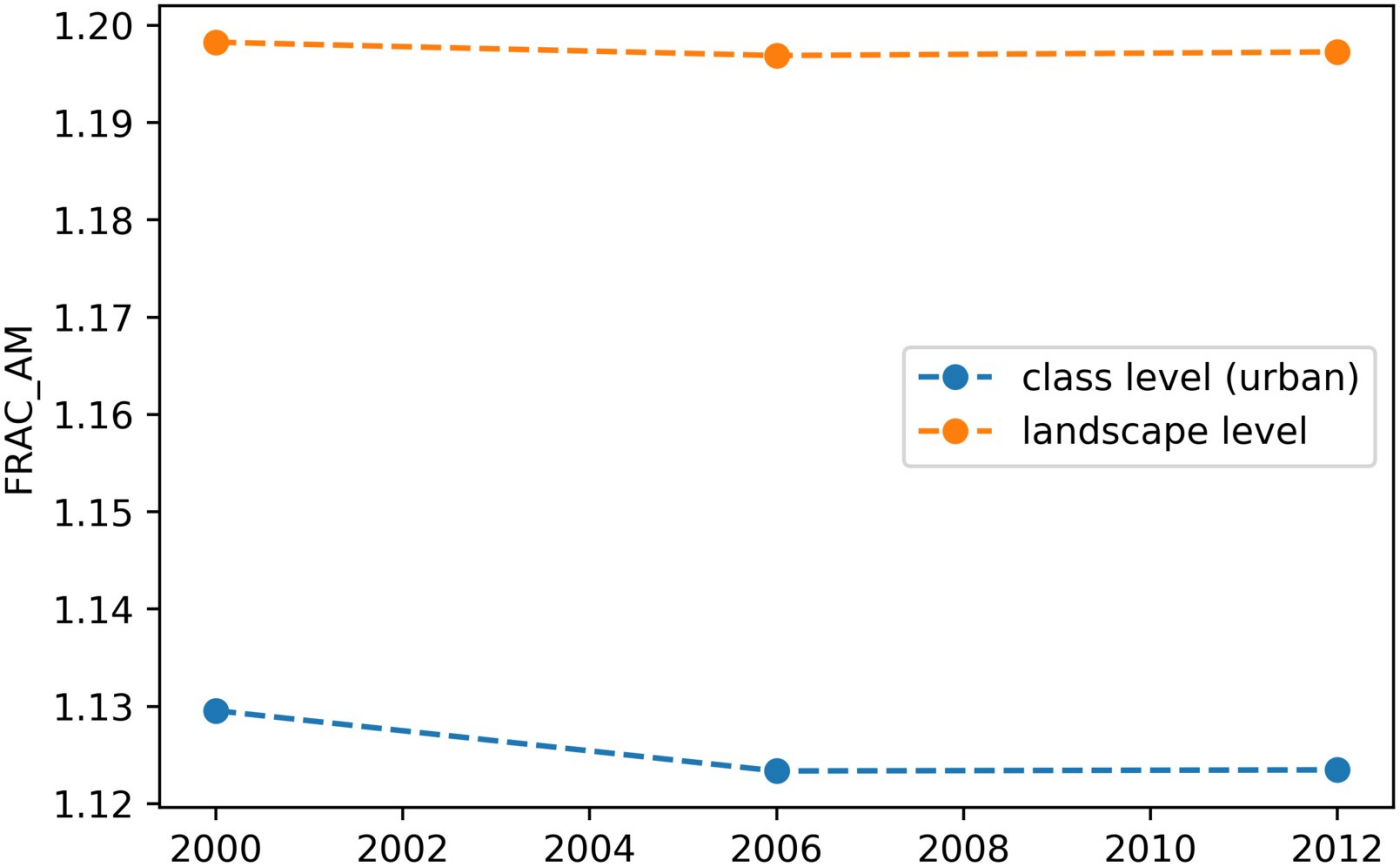
Example with a metric plotted at both the class and landscape level in a spatiotemporal analysis.

> ax.legend()

producing a plot as depicted in Fig 2.

In order to customize the resulting plot, the plot_metric method accepts, among other keyword arguments, a plt_kws keyword argument that will be forwarded to the matplotlib’s plot method (see the chapter 2 “Spatiotemporal analysis” of S1 Text).

## Zonal analysis

Landscape metrics are very sensitive to scale, that is, to the pixel resolution and especially to the spatial extent of the considered map [1, 12, 13]. To overcome such shortcoming, landscape ecologists often turn to methods of multiscale analysis which explicitly consider multiple scales, both in terms of resolution and map extents [14].

The PyLandStats library features two classes that might be used for such purpose. The first is BufferAnalysis, which segments a given landscape based on a series of buffers of increasing distances around a feature of interest, whereas the more generic ZonalAnalysis allows the user to freely choose how the landscape is segmented by providing a list of NumPy masks.

### Buffer analysis around a feature of interest

In line with the classic concentric models of location and land use, evaluating the spatial variation of the environmental characteristics across the urban-rural gradient has become one of the central topics of landscape ecology [15].

Consider a LULC raster file featuring a city and its rural hinterlands. Then, given a coordinate that represents the center of the feature of interest (e.g., a Shapely point with its coordinate reference system) and a list of buffer distances (in meters), a BufferAnalysis can be instantiated as follows:

> from shapely.geometry import Point

# latitude and longitude of the center of Lausanne in the OpenStreetMap

> base_mask = Point(6.6327025, 46.5218269)

> base_mask_crs = ‘*+proj = longlat +ellps = WGS84 +datum = WGS84 +no defs*’

# buffer distances (in meters)

> buffer_dists = [10000, 15000, 20000]

# instantiation of ‘BufferAnalysis’

**Fig 3:**
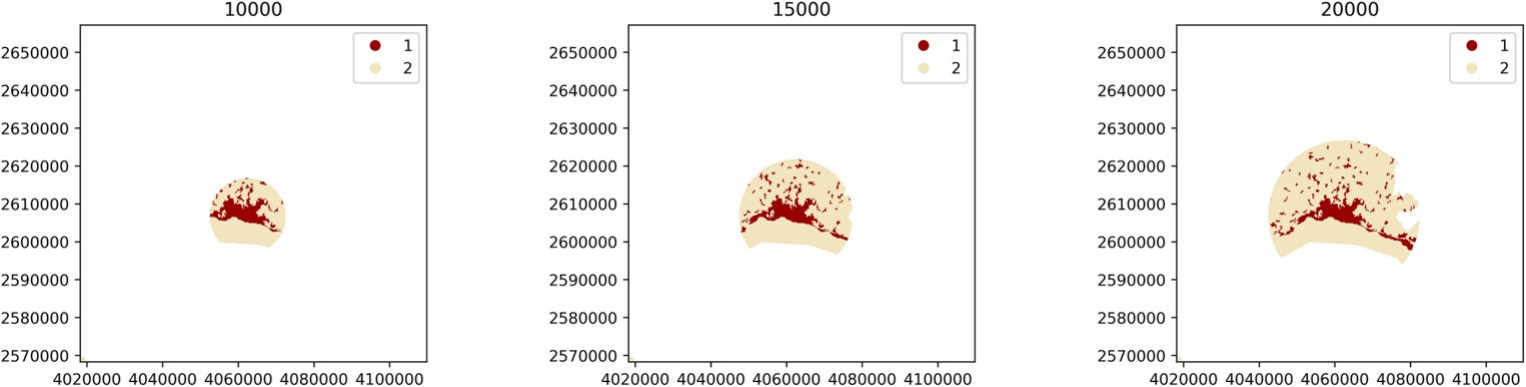
Landscapes generated by instantiating a BufferAnalysis with a raster of urban and non-urban LULC classes (values of 1 and 2 respectively), the coordinates of the city center as base mask, and buffer distances of 10000, 15000 and 20000m (corresponding to the three subplots from left to right).

> ba = pls.BufferAnalysis(

  path_to_raster, base_mask, buffer_dists, base_mask_crs = base_mask_crs)

where the BufferAnalysis instance will generate the landscape of interest for each buffer distance by masking the pixels of the input raster, as illustrated in Fig 3.

On the other hand, the base_mask argument might also be a polygon geometry (e.g., administrative boundaries) instead of a point. In such case, note that the list of buffer distances might start from zero in order to start computing the metrics for the region defined by the polygon geometry itself.

Like in the other classes, the data frames of class and landscape-level metrics can be obtained through the compute_class_metrics_df and compute_landscape_metrics_df methods respectively. For instance, the following snippet:

> ba.compute_class_metrics_df()

will return a data frame indexed by both the class value and buffer distance, as depicted in Table 8.

**Table 8:** Example data frame of class-level metrics for a buffer analysis.

class_val	buffer_dist	total_area	proportion_of_landscape	number_of_patches	patch_density	largest_patch_index	total_edge	…
1	10000	7261	24.9648	20	0.068764	21.5472	223900	…
	15000	9630	16.7106	46	0.0798223	11.5326	395200	…
	20000	12149	13.3476	76	0.0834981	7.30169	565200	…
2	10000	21824	75.0352	4	0.0137528	74.3614	223900	…
	15000	47998	83.2894	4	0.00694107	82.9493	395200	…
	20000	78871	86.6524	5	0.0054933	86.3151	565200	…

Again, the metrics that are considered in the analysis and how they metrics are computed can be customized by providing the metrics and metrics_kws keyword arguments respectively to the compute_class_metrics_df and compute_landscape_metrics_df methods, while the considered classes can be set as the classes keyword argument of compute_class_metrics_df.

**Fig 4:**
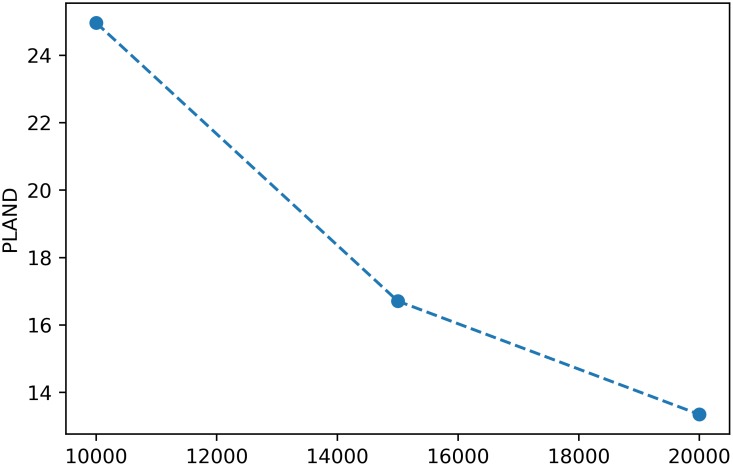
Example of a plot for a class-level metric in a buffer analysis. The x axis corresponds to the buffer distances.

On the other hand, and analogously to the SpatioTemporalAnalysis class, the metrics computed for each buffer distance in a BufferAnalysis instance can be plotted by means of the plot_metric method. Again, plot_metric takes an optional class_val keyword argument that if provided, plots the metric at the class level, and otherwise, plots the metric at the landscape level. For instance, the following snippet:

> ba.plot_metric(‘*proportion_of_landscape*’, class_val = 1)

will produce a plot for the metric at the class level as depicted in Fig 4.

**Fig 5:**
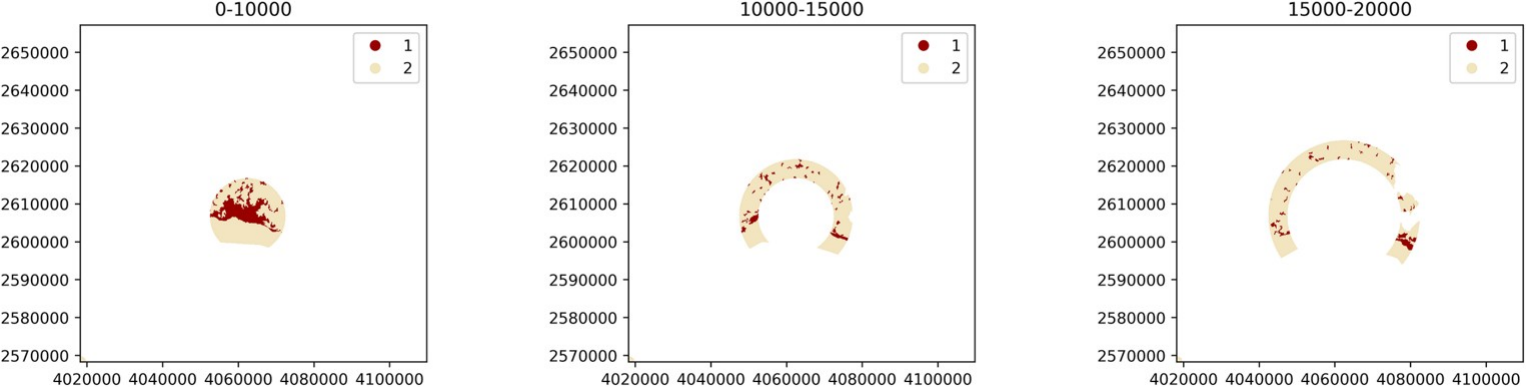
Landscapes generated by instantiating a BufferAnalysis with a raster of urban and non-urban LULC classes (values of 1 and 2 respectively), the coordinates of the city center as base mask, and buffer distances of 10000, 15000 and 20000m (corresponding to the three subplots from left to right) and buffer_rings set to True.

Another approach to examine how landscape patterns change across the urban-rural gradient is to compute the metrics for each buffer ring that defined between each pair of distances. For instance, for the buffer distances considered in latter example, i.e., 10000, 15000 and 20000, the metrics would be computed for the buffer rings that go from 0 to 10000 m, 10000-15000 m and 15000-20000 m. Such analysis can be performed in PyLandStats by setting the keyword argument buffer_rings to True, as in the snippet below:

> ba = pls.BufferAnalysis(

  input_filepath, base_mask, buffer_dists, base_mask_crs = base_mask_crs,

  buffer_rings = True)

where BufferAnalysis will generate the landscapes as depicted in Fig 5.

Under such circumstances, the buffer distance of each in the data frame of class and landscape-level metrics will be strings that represent the buffer distances that correspond to the start and end of each ring, as depicted in Table 9.

**Table 9:** Example data frame of class-level metrics for a buffer analysis computing the metrics for the buffer rings.

class_val	buffer_dist	total_area	proportion_of_landscape	number_of_patches	patch_density	largest_patch_index	total_edge	…
1	0-10000	7261	24.9648	20	0.068764	21.5472	223900	…
	10000-15000	2369	8.29976	37	0.129629	1.68518	168600	…
	15000-20000	2519	7.54372	37	0.110805	3.11152	169100	…
2	0-10000	21824	75.0352	4	0.0137528	74.3614	223900	…
	10000-15000	26174	91.7002	3	0.0105105	83.6282	168600	…
	15000-20000	30873	92.4563	8	0.0239578	76.117	169100	…

**Fig 6:**
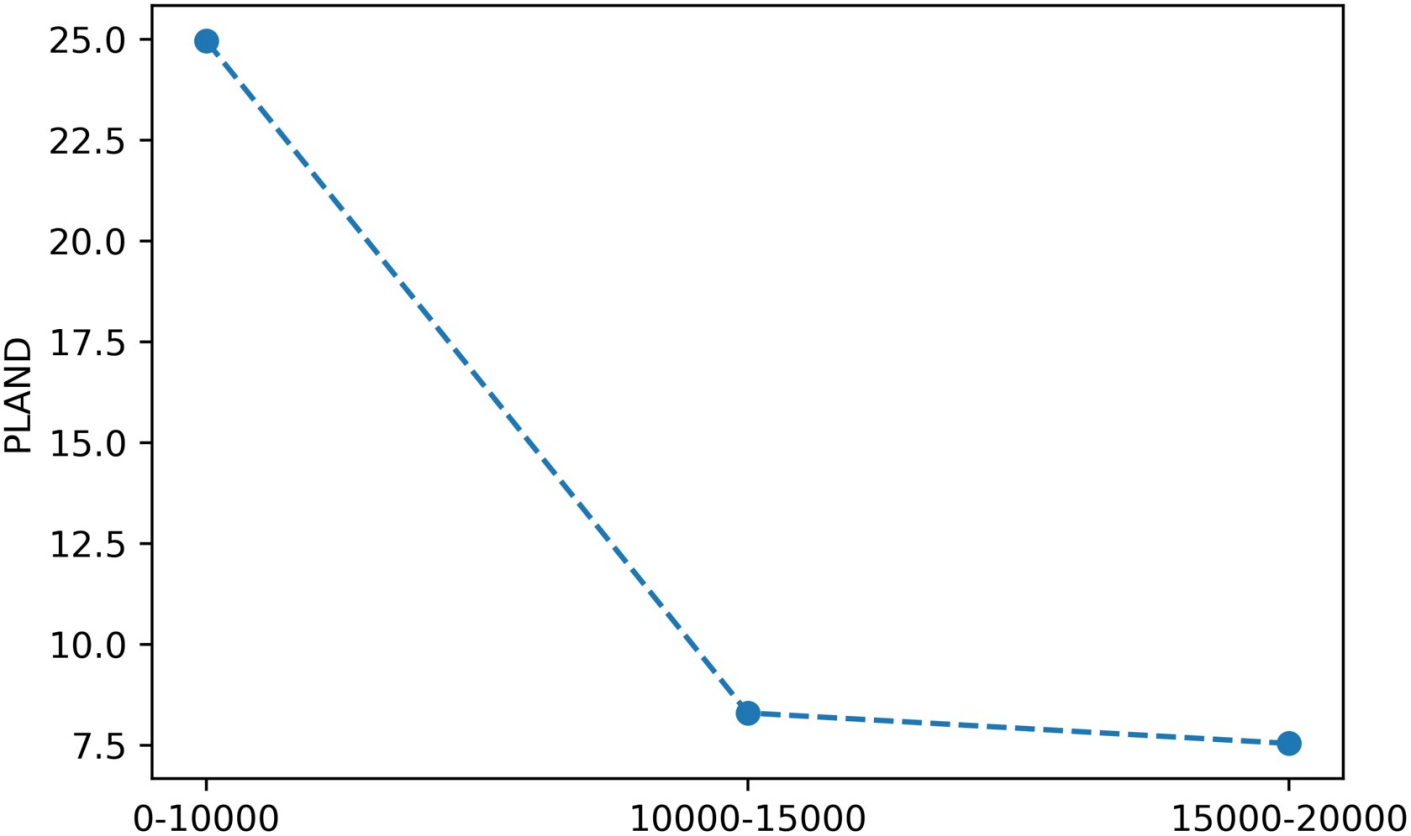
Example of a plot for a class-level metric in a buffer analysis that computes the metrics for the buffer rings. The x axis delineates three discrete points, each corresponding to a buffer ring, and whose label represents the ring’s start and end buffer distance.

Accordingly, the plot_metric method of a BufferAnalysis will produce a figure as depicted in Fig 6, where the x axis represents the buffer distances of the rings.

### Generic zonal analysis

In certain analysis cases, the user might consider more appropriate to compute the metrics along a decomoposition of the landscape different than concentric buffers, for example, rectangular transects. To that end, PyLandStats features the ZonalAnalysis class, which instead of a base mask, accepts a list of boolean arrays of the same shape of our landscape as masks to define our transects (or any other type of subregion). Consider the code snippet below:

# this reads the input raster landscape and creates a boolean base mask

# of the same shape of the landscape and filled with ‘False’ values

with rasterio.open(input_filepath) as src:

  base_mask_arr = np.full(src.shape, False)

masks_arr = []

# for a pixel resolution of 100m, this corresponds to transects of 30km

transect_len = 300

# this will iterate over three transects (0-30km, 30-60km, 60-90km)

for transect_start in range(0, 900, transect_len):

  mask_arr = np.copy(base_mask_arr)

  *# the 400 and 600 serve to slice the landscape vertically along the*

**Fig 7:**
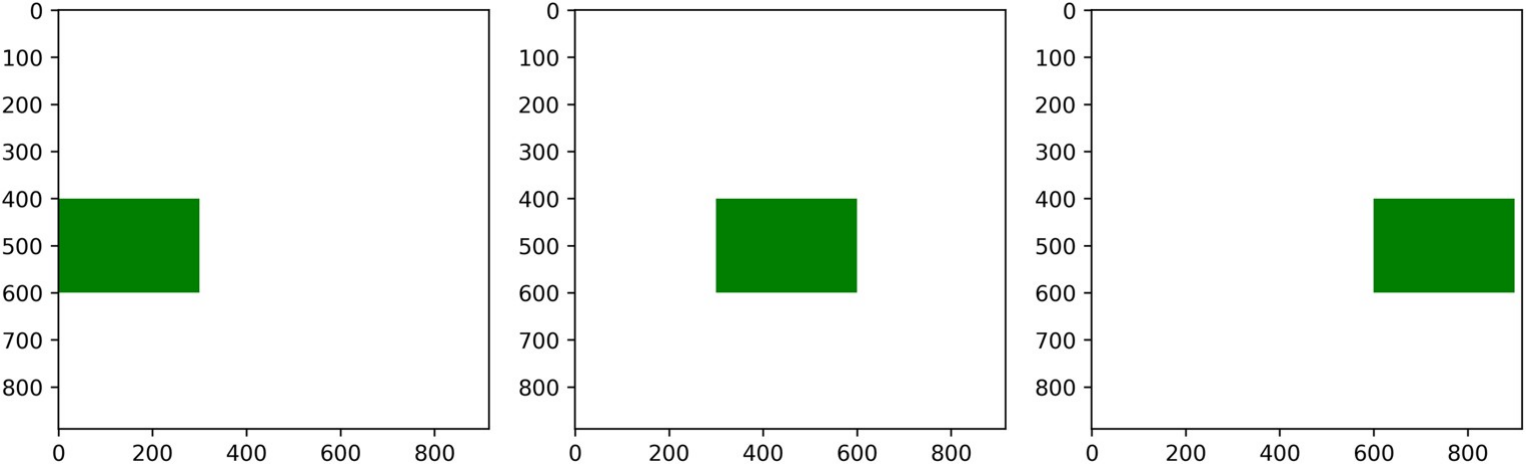
Example of a list of three boolean mask arrays that delineate three rectangular transects of a landscape.

  *# 20km where the feature of interest is located*

  mask_arr[400:600,transect_start:transect_start+transect_len] = True

  masks_arr.append(mask_arr)

where the variable masks_arr will be a list of three NumPy boolean arrays, each corresponding to a distinct rectangular transect, as plotted in Fig 7.

The instantiation of ZonalAnalysis requires the list of mask arrays (e.g., the masks_arr variable created above) as second argument. Additionally, the keyword argument attribute_values might be used to map an identifying value or label to each of our landscapes. In this example, a list of strings will be provided in a form which denotes that each landscape corresponds to the transect from kilometers 0 to 30, 30 to 60 and 60 to 90 respectively:

> attribute_values = [‘*0-30*’, ‘*30-60*’, ‘*60-90*’]

**Fig 8:**
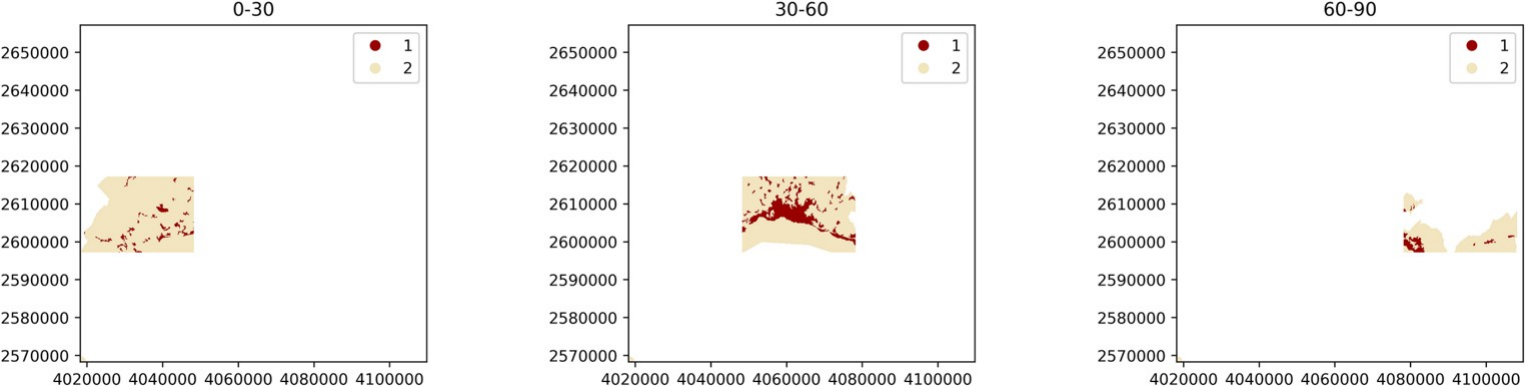
Landscapes generated by instantiating a ZonalAnalysis for three rectangular transects.

> za = pls.ZonalAnalysis(

  input_filepath, masks_arr, attribute_values = attribute_values)

where ZonalAnalysis will generate the landscapes as depicted in Fig 8.

In ZonalAnalysis instances, the data frames of metrics are indexed by the values provided to the keyword argument attribute_values as depicted in Table 10.

**Table 10:** Example data frame of class-level metrics in a zonal analysis of three transects.

class_val	attribute_values	total_area	proportion_of_landscape	number_of_patches	patch_density	largest_patch_index	total_edge	…
1	0-30	2641	5.0768	37	0.0711251	0.707407	216700	…
	30-60	9577	17.6965	40	0.0739126	12.2806	370500	…
	60-90	1761	9.27281	9	0.0473909	6.90854	71900	…
2	0-30	49380	94.9232	2	0.0038446	94.9194	216700	…
	30-60	44541	82.3035	6	0.0110869	81.8859	370500	…
	60-90	17230	90.7272	6	0.0315939	53.2199	71900	…

Again, the data frames of metrics ZonalAnalysis can also customized by providing the metrics and metrics_kws keyword arguments to the compute_class_metrics_df and compute_landscape_metrics_df methods, and additionally by the classes keyword argument in compute_class_metrics_df.

**Fig 9:**
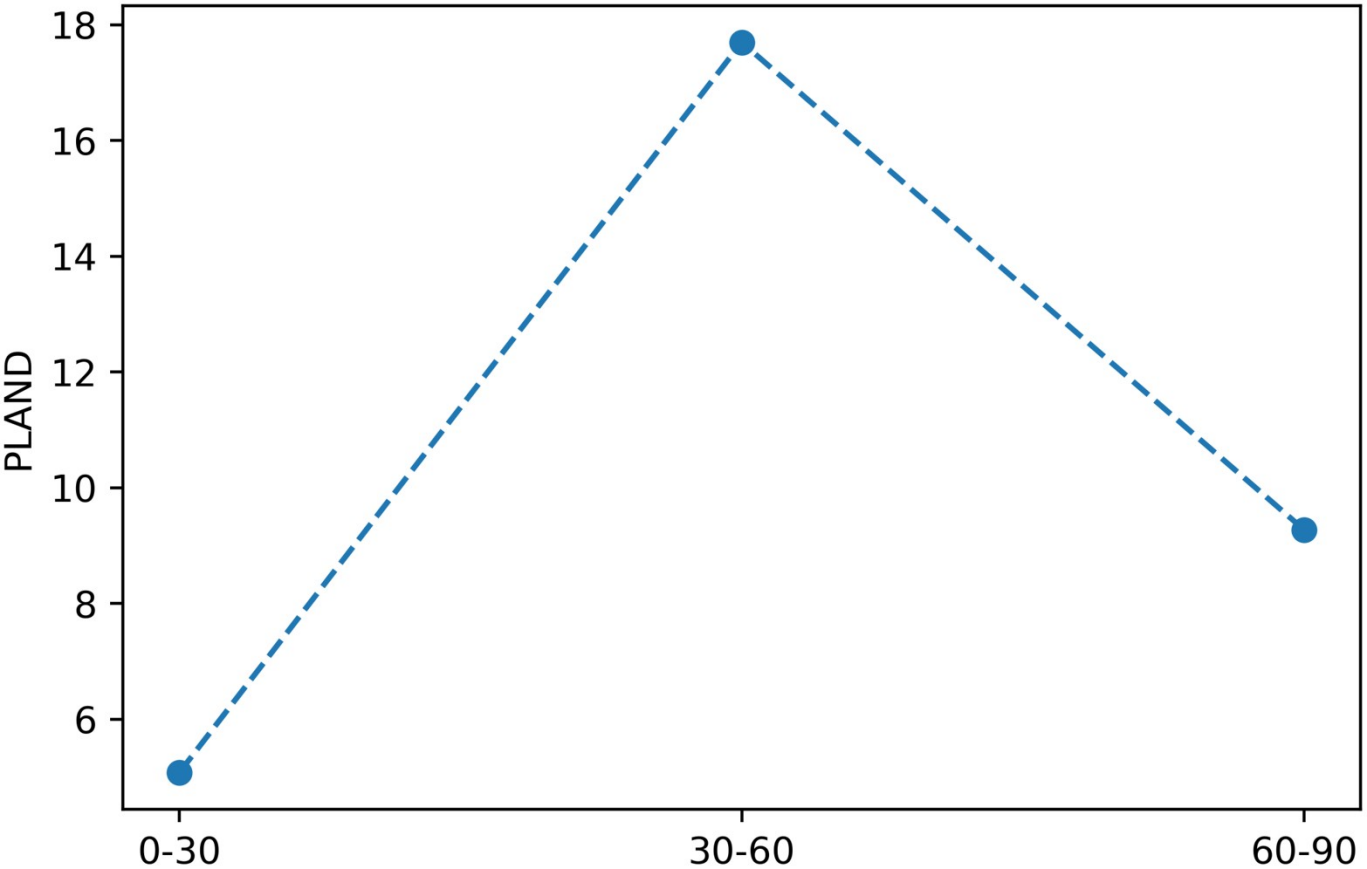
Example of a plot for a class-level metric in a zonal analysis of three transects. The x axis corresponds to the values provided to the keyword argument attribute_values provided to the initialization of ZonalAnalysis.

In order to plot a metric’s computed value for each subregion, the class ZonalAnalysis features a plot_metric method which works in the same way as its counterpart in SpatioTemporalAnalysis and BufferAnalysis. For instance, the following snippet:

> za.plot_metric(‘*proportion_of_landscape*’, class_val = 1)

will produce a plot for the metric at the class level as depicted in Fig 9.

## Spatiotemporal buffer analysis

The zonal analysis methods presented above are themselves multiscale analysis approaches since they explicitly consider multiple map extents. Accordingly, the BufferAnalysis and ZonalAnalysis classes might be employed to obtain scalograms, namely, response curves of the metrics to changing the map extent [16].

Nevertheless, when performing spatiotemporal analyses, it might also be useful to evaluate how the computed time series of metrics responds to changes in the map extent. To that end, PyLandStats features an additional SpatioTemporalBufferAnalysis class, which is instantiated like a BufferAnalysis except that the first argument is a temporally-ordered list of landscape raster snapshots—like in the SpatioTemporalAnalysis class—instead of a single raster landscape. In addition, like the SpatioTemporalAnalysis class, a list with the dates that correspond to each of the landscape snapshots can be passed to the keyword argument dates. Putting it all together, SpatioTemporalBufferAnalysis can be instantiated as in:

# Note: ‘input_filepaths’ is a list (like in ‘SpatioTemporalAnalysis’)

> stba = pls.SpatioTemporalBufferAnalysis(

  input_filepaths, base_mask, buffer_dists,

  base_mask_crs = base_mask_crs, dates = [2000, 2006, 2012])

Like BufferAnalysis, a SpatioTemporalBufferAnalysis can also be instantiated from a polygon geometry. The data frame of class and landscape-level metrics can be computed by means of the the compute_class_metrics_df and compute_landscape_metrics_df methods respectively, which again, might also be customized by providing the metrics and metrics_kws keyword arguments, and additionally by the classes keyword argument in compute_class_metrics_df. In SpatioTemporalBufferAnalysis instances, the data frames are indexed by the buffer distances and the snapshot dates (and also by the LULC class values in the class-level data frame, as depicted in Table 11).

**Table 11:** Example data frame of class-level metrics in a spatiotemporal buffer analysis.

buffer_dist	class_val	dates	total_area	proportion_of_landscape	number_of_patches	patch_density	largest_patch_index	total_edge	…
10000	1	2000	7261	24.9648	20	0.068764	21.5472	223900	…
		2006	7205	24.7722	20	0.068764	21.0211	226600	…
		2012	7205	24.7722	20	0.068764	21.0211	227000	…
	2	2000	21824	75.0352	4	0.0137528	74.3614	223900	…
		2006	21880	75.2278	4	0.0137528	74.5539	226600	…
		2012	21880	75.2278	4	0.0137528	74.5539	227000	…
15000	1	2000	9630	16.7106	46	0.0798223	11.5326	395200	…
		2006	9278	16.0998	49	0.0850281	11.2671	391300	…
		2012	9320	16.1727	50	0.0867634	11.2671	395500	…
	2	2000	47998	83.2894	4	0.00694107	82.9493	395200	…
		2006	48350	83.9002	4	0.00694107	83.5601	391300	…
		2012	48308	83.8273	4	0.00694107	83.4872	395500	…
20000	1	2000	12149	13.3476	76	0.0834981	7.30169	565200	…
		2006	11827	12.9938	78	0.0856955	7.1336	566200	…
		2012	11882	13.0543	79	0.0867941	7.1336	571400	…
	2	2000	78871	86.6524	5	0.0054933	86.3151	565200	…
		2006	79193	87.0062	6	0.00659196	86.6678	566200	…
		2012	79138	86.9457	7	0.00769062	86.604	571400	…

**Fig 10:**
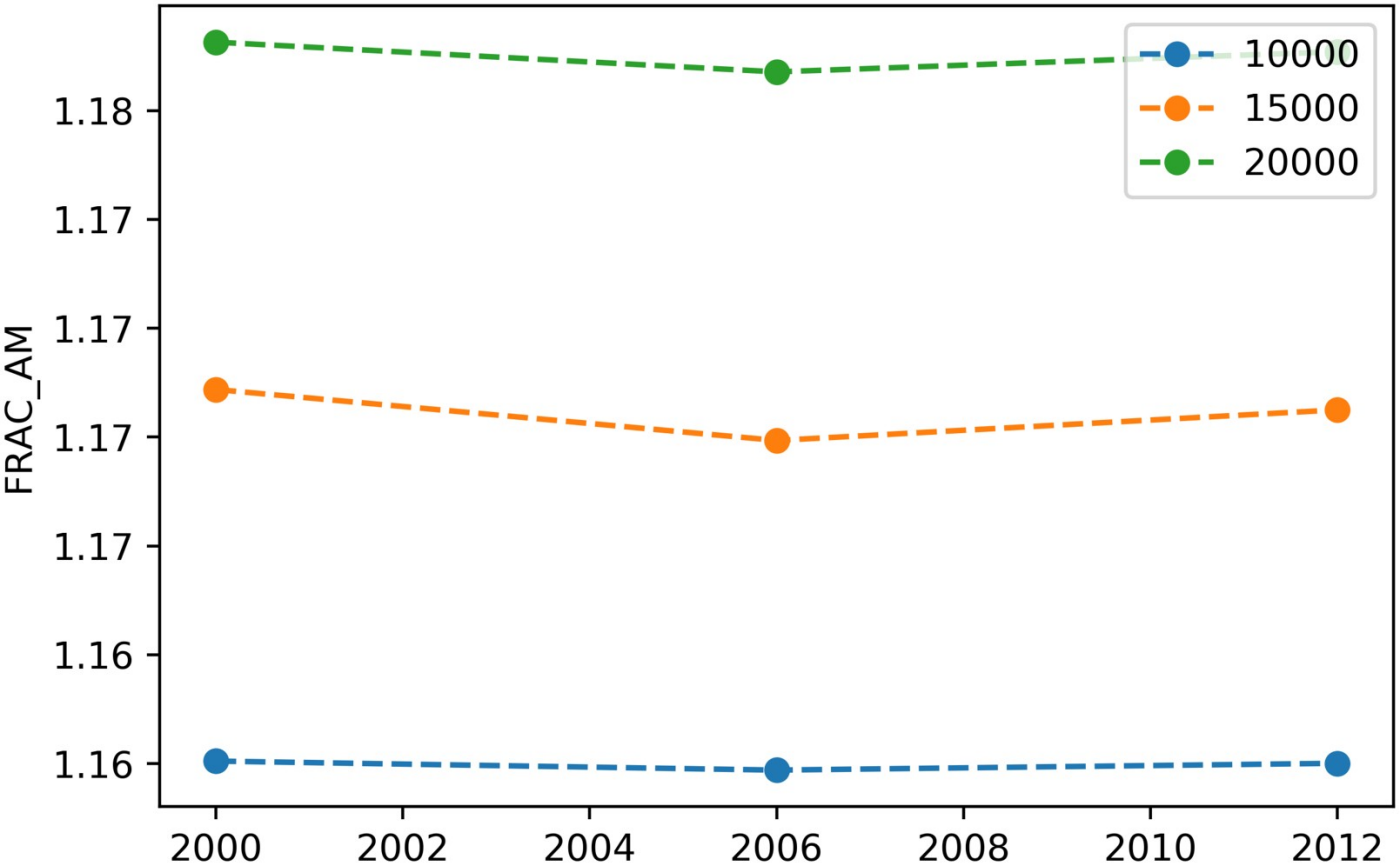
Example of a plot for a landscape-level metric in a spatiotemporal buffer analysis.

The SpatioTemporalBufferAnalysis class features a plot_metric method with the same signature of its counterparts in SpatioTemporalAnalysis, BufferAnalysis and ZonalAnalysis. For example, the snippet below:

> stba.plot_metric(‘*fractal_dimension_am*’)

will plot the temporal evolution of the area-weighted fractal dimension at the landscape level for the three buffer distances in the same axis, producing an output as depicted in Fig 10.

Although this is beyond the scope of this article, the above plot suggests that the area-weighted fractal dimension shows a predictable response to changing the spatial extent of the considered landscape [16, 17].

## The PyLandStats library

### Availability and installation

The source code of PyLandStats is available in a GitHub repository at https://github.com/martibosch/pylandstats, and is licensed under the open source GNU General Public License 3 (GNU GPLv3) to ensure that any derivative work is kept as open source. The easiest way to install PyLandStats is by installing the dedicated conda recipe hosted on the conda-forge channel at https://anaconda.org/conda-forge/pylandstats, as in:

$ conda install -c conda-forge pylandstats

The above command will install all the necessary requirements to run all the features of PyLandStats. Alternatively, a dedicated Python package is hosted on the Python Package Index (PyPI) at https://pypi.org/project/pylandstats/, and can be readily installed with pip as in:

$ pip install pylandstats

Nevertheless, the BufferAnalysis and SpatioTemporalBufferAnalysis classes have dependencies that cannot be installed with pip, namely the Geospatial Data Abstraction Library (GDAL) and the Geometry Engine Open Source (GEOS). In order to use these two PyLandStats classes, GDAL and GEOS must be present at the time of installing PyLandStats, which in this case will further require specifying the geo extra requirements as in:

$ pip install pylandstats[geo]

Unit tests are run within the Travis Continuous Integration (Travis CI) platform at https://travis-ci.org/martibosch/pylandstats every time that new commits ar pushed to the GitHub repository. Additionally, test coverage is reported on Coveralls at https://coveralls.io/github/martibosch/pylandstats?branch=master.

The documentation of PyLandStats is hosted in Read the Docs at https://pylandstats.readthedocs.io/ and is also available in S1 Text. Additionally, a collection of example notebooks with a thorough overview of PyLandStats’s features is provided at a dedicated GitHub repository at https://github.com/martibosch/pylandstats-notebooks, which can be executed interactively online by means of the Binder web service [18]. Such repository includes unit tests which ensure the correctness of the computations (see S5 Code).

Finally, an example application of PyLandStats in an academic article can be found in the analysis of the spatiotemporal patterns of urbanization of three Swiss urban agglomerations by Bosch and Chenal [19], and all the code and materials necessary to reproduce the results are available in a dedicated GitHub repository at https://github.com/martibosch/swiss-urbanization.

### Dependencies and implementation details

The PyLandStats package is fully implemented in Python, and requires the Python packages NumPy, SciPy, pandas, matplotlib, rasterio. The first four are among the most popular packages for scientific and data-centric Python and are used for a wide-variety of scientific needs, whereas rasterio is a popular library to read and write geospatial raster data. In PyLandStats, NumPy arrays are used to represent landscapes and patch-level metrics. In addition, NumPy functions are used in the computations of all the implemented landscape metrics. The SciPy library is used to segment the patches in the landscape arrays, compute the inter-patch nearest-neighbor distances, and to compute the coefficient of variation of the patch-level landscape metrics. The pandas data frames are used to build the data frames of landscape metrics, matplotlib is used to produce the plots and rasterio is used to read raster data, plot the landscapes as well as to rasterize the vector geometries used in BufferAnalysis and SpatioTemporalBufferAnalysis. As noted above, the foregoing two classes further require the GeoPandas and Shapely Python packages.

The implementation of PyLandStats is organized in Python modules, where the classes described throughout this paper are defined. Such object-oriented design offers many advantages. On the one hand, it allows both for a conceptual separation and reusability of the functionalities, which enhances the maintainability and extensibility of PyLandStats. On the other hand, Python properties serve to cache results that are computationally expensive to obtain, which can later be accessed in constant (almost immediate) time. This mechanism is exploited to cache intermediate results that are later used to compute the metrics (see S6 Code). More precisely, instances of the Landscape class cache the list of patches, each with its respective LULC class, area, perimeter and nearest-neigbhor distance, as well as the pixel adjacency matrix, i.e., the number of adjacencies between pixels of each landscape class (including adjacencies between pixels of the same class). Furthermore, such mechanism eases the task of implementing new metrics, since the vast majority of landscape metrics found throughout the academic literature can be straight-forwardly computed out of such cached properties (see the section 1.1 “List of implemented metrics” of S1 Text as well as [5]). Finally, as follows from the cache mechanism described above, the memory size of a Landscape instance scales linearly with the number of patches present in the respective raster landscape.

Regarding the performance, the most expensive operations of PyLandStats are the computation of the adjacency matrix, and more importantly, the computation of the inter-patch nearest-neighbor distances. The code for the former is transformed from Python to C++ by means of the Pythran ahead-of-time compiler [20], which achieves speed-ups of an order of magnitude of three. The code for the latter consists of a slow nested Python loop that iterates over each patch of each class and employs SciPy’s implementation of the K-d tree in Cython [21] in order to find the nearest neigbor of each patch. The computation of the inter-patch nearest-neighbor distances is by far the main performance bottleneck of PyLandStats (see S6 Code), and it is therefore recommended that in analysis cases that do not require computing euclidean nearest-neighbor metrics avoid its computation by making use of the metrics keyword argument as explained above.

### Improvements of PyLandStats over existing software packages

There have been many other freely-available software packages to compute landscape metrics [6] (see Table 12). By far, the most popular one has been FRAGSTATS [22], yet as a stand-alone software, its functions cannot be directly integrated into advanced computational workflows. Furthermore, FRAGSTATS is not open-source software. Recently, the open-source R package landscapemetrics [23] has been developed to overcome such shortcomings by relying on a well-established spatial framework in R. On the other hand, the only available tool to compute landscape metrics in Python is the LecoS package [24], which is designed as a QGIS plugin.

**Table 12:** Comparison of FRAGSTATS, landscapemetrics, LecoS and PyLandStats.

Characteristic	FRAGSTATS	landscapemetrics	LecoS	PyLandStats
open source	no	yes	yes	yes
programming language	?	R	Python	Python
cross-platform compatibility	no	yes	yes	yes
integration into advanced workflows	no	yes	QGIS only	yes
Benchmark Vaud [s]	0.61	14.27	-	0.91
Benchmark Bern and Valais [s]	33.31	553.45	-	32.2

The two benchmarks consist in the computation of the 95 metrics implemented in PyLandStats for the landscape snapshots of the canton of Vaud (889x916 pixels of 2 LULC classes) and the cantons of Bern and Valais (1640x1319 pixels of 28 LULC classes) respectively (see S6 Code for more details). Both landscapes have been derived from the Corine Land Cover [7] dataset for the year 2000. Note that LecoS has been excluded from the benchmarks since only features 20 landscape metrics.

The computed values for the landscape metrics in PyLandStats are the same as in FRAGSTATS, with a maximum relative difference of 0.1% (see S5 Code). Furthermore, the performance of both packages is very similar. Nevertheless, unlike FRAGSTATS, PyLandStats is open source and it is therefore straightfoward for users to contribute to its development on its GitHub repository. On the other hand, PyLandStats is an alternative to landscapemetrics for those users that prefer to write their computational workflows in Python rather than R. Additionally, the cache mechanisms included within PyLandStats lead to significantly better performance and make it more suitable for experimentation in interactive environments such as Jupyter notebooks [25], since it ensures that the marginal cost of subsequent calls to compute a metric are minimal (see S6 Code).

Finally, although LecoS is based on the NumPy and SciPy stack (like PyLandStats), only 20 metrics have been implemented, and its design as a QGIS plugin forces the users to adapt the computational workflows to QGIS. In sharp contrast, PyLandStats is designed as a Python package which can be directly used in Python scripts, Jupyter notebooks and in other Python packages including QGIS plugins.

In view of the growing popularity of Jupyter notebooks and continuous releases of new Python packages to visualize geospatial data interactively, it is reasonable to expect that geospatial scientists, including landscape ecologists, will increasingly turn to the Jupyter environments for their analyses. From this perspective, PyLandStats intends to offer a Python package that geospatial scientists can use in order to compute landscape metrics, and whose modularity and object-oriented design allows it to evolve and adapt to new developments in the Python and Jupyter ecosystem.

## Supporting information

S1 Text PyLandStats documentation. (PDF)

S1 Table Table of metrics implemented in PyLandStats. (PDF)

S1 Code Landscape analysis with PyLandStats for the canton of Vaud (Switzerland), as Jupyter notebook. (IPYNB)

S2 Code Spatiotemporal analysis with PyLandStats for the canton of Vaud (Switzerland), as Jupyter notebook. (IPYNB)

S3 Code Zonal analysis with PyLandStats for the canton of Vaud (Switzerland), as Jupyter notebook. (IPYNB)

S4 Code Spatiotemporal buffer analysis with PyLandStats for the canton of Vaud (Switzerland), as Jupyter notebook. (IPYNB)

S5 Code Comparison of the metrics computed in FRAGSTATS v4 and PyLandStats for the canton of Vaud (Switzerland), as Jupyter notebook. (IPYNB)

S6 Code Performance notes and benchmarks comparing FRAGSTATS v4, landscapemetrics and PyLandStats, as Jupyter notebook. (IPYNB)
