# Supplementary material for: PyLandStats: An open-source Pythonic library to compute landscape metrics
Source: PLoS One. 2019 Dec 5;14(12):e0225734. doi: 10.1371/journal.pone.0225734 (PMC6894873; doi:10.1371/journal.pone.0225734)
Supplement: S1 Table — (PDF) [file pone.0225734.s002.pdf]

Table S1. Metrics implemented in PyLandStats.

Metric name	PyLandStats label	Level	Description
Area	`area`	patch, class, landscape	Area of each landscape patch
Perimeter	`perimeter`	patch, class, landscape	Perimeter of each landscape patch
Perimeter/area ratio	`perimeter_area_ratio`	patch, class, landscape	The ratio between the perimeter and area of each patch of the landscape
Shape index	`shape_index`	patch, class, landscape	A measure of shape complexity, similar to the perimeter-area ratio, but correcting for its size problem by adjusting for a standard square shape
Fractal dimension	`fractal_dimension`	patch, class, landscape	A measure of shape complexity appropriate across a wide range of patch sizes
Euclidean nearest neighbor	`euclidean_nearest_neighbor`	patch, class, landscape	Distance to the nearest neighboring patch of the same class based on the shortest edge-to-edge distance
Total area	`total_area`	class, landscape	Total area
Proportion of landscape	`proportion_of_landscape`	class	Measures the proportional abundance of a particular class within the landscape
Number of patches	`number_of_patches`	class, landscape	Number of patches
Patch density	`patch_density`	class, landscape	Density of class patches, which is arguably more useful than the number of patches since it facilitates comparison among landscapes of different sizes
Largest patch index	`largest_patch_index`	class, landscape	The proportion of total landscape comprised by the largest patch
Total edge	`total_edge`	class, landscape	Measure of the total edge length
Edge density	`edge_density`	class, landscape	Measure of edge length per area unit, which facilitates comparison among landscapes of different sizes
Landscape shape index	`landscape_shape_index`	class, landscape	Measure of class aggregation that provides a standardized measure of edginess that adjusts for the size of the landscape
Contagion	`contagion`	landscape	Measure of aggregation that measures the probability that two random adjacent cells belong to the same class
Shannon's diversity index	`shannon_diversity_index`	landscape	Measure of diversity that reflects the number of classes present in the landscape as well as the relative abundance of each class

The column "PyLandStats label" displays the snake case representation of the metric name, which corresponds to the label to refer to each metric within the PyLandStats' classes and methods (see also the documentation of PyLandStats at `https://pylandstats.readthedocs.io/` or in S1 Text). The horizontal rules separate the three main groups of metrics, namely those that provide a scalar value for each patch of the landscape (i.e., patch-level metrics), those that provide a scalar value that aggregates a characteristic of interest over all patches of a given LULC class (i.e., class-level metrics), and those that aggregate a characteristic of interest over all the patches of a landscape (i.e., landscape-level metrics). Additionally, like FRAGSTATS, PyLandStats features six distribution-statistics metrics for each patch-level metric, which consist in a statistical aggregation of the values computed for each patch of a class or the whole landscape:

- the mean, which can be computed by adding a `_mn` suffix to the method name, e.g., `area_mn`
- the area-weighted mean, which can be computed by adding an `_am` suffix to the method name, e.g., `area_am`
- the median, which can be computed by adding a `_md` suffix to the method name, e.g., `area_md`
- the range, which can be computed by adding a `_ra` suffix to the method name, e.g., `area_ra`
- the standard deviation, which can be computed by adding a `_sd` suffix to the method name, e.g., `area_sd`
- the coefficient of variation, which can be computed by adding a `_cv` suffix to the method name, e.g., `area_cv`

Note that the distribution-statistics metrics do not appear in the table below.
