# Supplementary material for: PyLandStats: An open-source Pythonic library to compute landscape metrics
Source: PLoS One. 2019 Dec 5;14(12):e0225734. doi: 10.1371/journal.pone.0225734 (PMC6894873; doi:10.1371/journal.pone.0225734)
Supplement: Attachment — Submitted filename: rebuttal_letter.pdf [file pone.0225734.s009.pdf]

Rebuttal letter

Editor

In order to avoid any misunderstandings, the “Acknowledgements” section has been dropped. The funding statement can be left as it is since the authors have not received any specific funding for this work.

Reviewer #1

The metrics have been listed in Table S1. The choice of implemented metrics has been mostly guided by their recurrence in the studies of land use/land cover change associated to urbanization. As detailed in the section “The PyLandStats library”, the organization of the code and its open-source model enable users to contribute to the repository by requesting the implementation of new metrics or actually implementing them. On the other hand, lines 68-74 do not intend to suggest which metrics should be selected, but instead to show that users might employ the metrics keyword argument in order to limit their analysis to a subset of metrics of their choice.

Two benchmarks comparing the execution times with those of FRAGSTATS and landscapemetrics has been included as Code S6. Such supporting information also features further details regarding the performance of PyLandStats. Additionally, a note regarding the memory size of Landscape objects has been added to the section “Dependencies and implementation details”

The values computed for all the metrics implemented in PyLandStats are compared with those computed with FRAGSTATS v4 in Code S5, showing a maximum discrepancy of 0.1%. Such notebook is included in the example notebook repository (https://github.com/martibosch/pylandstats-notebooks), which is also subject to automated tests in Travis CI. Therefore, the automated tests for such repository do test for the correctness of the computations.

I have updated the figures with higher DPI and updated the color palette of the landscape raster plots.

Properties were used to compute the data frames of metrics in spatiotemporal, buffer and gradient analysis in order to avoid recomputing the metrics each time that the user desired to obtain a data frame or plot. Nevertheless, after the in-depth exploration of the performance detailed in Code S6, it seems that such performance gains are negligible. Therefore, in order to ensure a consistent API, the computation of data frames of metrics will use methods (instead of properties) throughout all the classes of PyLandStats.

I have added a note (and updated the notebook in Code S2) to acknowledge that dates might also be specified as strings or datetime objects.

The generic GradientAnalysis class has been renamed to ZonalAnalysis, and the manuscript and notebook of Code S3 have updated accordingly.

A reference to matplotlib has been added (with its corresponding citation). For consistency, the citations for numpy and pandas have also been added (note that scipy, rasterio, shapely and geopandas do not have proper academic articles).

I believe that after the unification of the API regarding the computation of data frames with methods, it is no longer necessary to clarify when metrics are computed since they are indeed computed when the methods are called. Additionally, details regarding how metrics are computed in PyLandStats are covered in section “Dependencies and implementation details” as well as Code S6.

I have corrected the typos.

Reviewer #2

I have updated the figures with higher DPI and updated the color palette of the landscape raster plots.

Two benchmarks comparing the execution times with those of FRAGSTATS and landscapemetrics has been included as Code S6. Such supporting information also features further details regarding the performance of PyLandStats.

The values computed for all the metrics implemented in PyLandStats are compared with those computed with FRAGSTATS v4 in Code S5, showing a maximum discrepancy of 0.1%.

The link to the GitHub repository is displayed correctly in the manuscript (https://github.com/martibosch/pylandstats) and I believe that it has been working all along.

Regarding the incompatibility between rasterio and GDAL’s Python bindings, I have never encountered any issues, neither when installing PyLandStats via pip nor when installing it via conda. In any case, users might report any of such eventual issues in the GitHub repository.
